# Phage therapy: A novel approach against multidrug-resistant pathogens

**DOI:** 10.1007/s13205-024-04101-8

**Published:** 2024-09-30

**Authors:** Arushi Kapoor, Samriti Balaji Mudaliar, Vyasraj G. Bhat, Ishita Chakraborty, Alevoor Srinivas Bharath Prasad, Nirmal Mazumder

**Affiliations:** 1 Robert R Mcormick School of Engineering and Applied Science, Northwestern University, Illinois, USA; 2 Department of Public Health Genomics, Manipal School of Life Sciences, Manipal Academy of Higher Education, Manipal, Karnataka 576104 India; 3 Department of Biophysics, Manipal School of Life Sciences, Manipal Academy of Higher Education, Manipal, Karnataka 576104 India

**Keywords:** Bacteriophage, Phage therapy, Bacterial disease, Drug-resistant pathogens, Antimicrobial resistance

## Abstract

The rapid rise of multidrug-resistant (MDR) organisms has created a critical need for alternative treatment options. Phage therapy is gaining attention as an effective way to fight bacterial infections by using lytic bacteriophages to specifically target and kill harmful bacteria. This review discusses several phage therapeutic options and emphasizes new developments in phage biology. Phage treatment has proven to be successful against MDR bacteria, as evidenced by multiple human clinical trials that indicate favorable results in treating a range of diseases caused by these pathogens. Despite these promising results, challenges such as phage resistance, regulatory hurdles, and the need for standardized treatment protocols remain. To effectively combat MDR bacterial infections, future research must focus on enhancing phage effectiveness, guaranteeing safety for human usage and incorporating phage therapy into clinical practice.

## Introduction

In recent years, the emergence of multidrug-resistant (MDR) bacteria has led to a pressing need to explore the potential role of phage therapy as an alternative treatment. For patients suffering from infections caused by pathogenic MDR bacteria, the options for treatment are quite limited. New mechanisms of resistance are emerging and spreading globally, leading to the inability to treat infectious diseases that ultimately result in disability and death. The discovery of newer antibiotics is laborious and complex; thus, new alternatives such as phage therapy are attracting renewed interest (Lin et al. 1857). Estimates reveal that by 2050, approximately 444 million people will succumb to infections, and birth rates will decline rapidly (Aslam et al. 2018). The development and spread of multidrug resistance in bacteria can be due to several mechanisms. The indiscriminate use of antibiotics by populations of developing countries such as Indonesia, Thailand, China, and India are one of the contributing factors (Hosain et al. 2021). According to a study conducted in Thailand, 43% of deaths caused by hospital-acquired MDR bacterial infections in 2010 were excess mortality related to MDR (Lim et al. 2016). Some of the countries with the highest numbers of hospital associated antibiotic resistant infections per year are China with 52 million and India with 9 million (Balasubramanian et al. 2023). In India, it is estimated that by the year 2050 about 2 million deaths would occur due to antimicrobial resistance. In India, Gram-negative and Gram-positive bacteria have high resistance to fluoroquinolones, carbapenem, and colistin. High resistance to even newer antimicrobials such as carbapenems and faropenem has been documented. Pathogens such *Salmonella typhi*, *Shigella*, *Pseudomonas*, and *Acinetobacter* have been found to be highly resistant to fluoroquinolones and cephalosporins (third generation) (Chandra et al. 2020). In Indonesia from data collected from 20 hospitals, it was seen that there is a growth in the percentage of bacterial species *E.coli* and *K.pneumoniae* that are resistant to antibiotics such as carbapenems, fluoroquinoles and third generation cephalosporins. It was also noted that about 15% of patients in the hospitals experienced healthcare associated infections sustained my multidrug resistant microorganisms (Siahaan et al. 2023). Antibiotic resistance also leads to a large economic burden globally. In the Southeast Asian region, antibiotics are used without proper prescription from doctors, which leads to the development of drug-resistant microorganisms (Nepal and Bhatta 2018). Antimicrobial resistance has become a global concern and is no longer restricted to a particular region. Some of the factors leading to the global spread of AMR are global travel, trade and migration. The overuse of antibiotics in agriculture and animal husbandry has led to the development of resistant microorganisms all over the world (Prestinaci et al. 2015). It is estimated that the economic loss caused by antimicrobial resistance would be approximately $300 billion to about $1 trillion by the year 2050 (Burki 2018).

The projected cost of infections caused by MDR bacteria is roughly $55 billion in the US and €1.5 billion in the EU each year. By 2050, the cost is projected to increase to $110 trillion (Dadgostar 2019). In light of these statistics, bacteriophage therapy is assumed to have increased importance. Bacteriophage therapy involves the use of virulent phages against bacteria. Bacteriophages are viruses that can infect and replicate within bacterial cells. There are two primary life cycles of bacteriophages: the virulent or obligate lytic phage cycle, in which phages infect and kill cells quickly, and the lysogenic phage cycle, in which lysogenic phages integrate into the host genome or enter the lytic life cycle and cause lysis of the cell (Jamal et al. 2019).

Although phage therapy provides a viable alternative for the treatment of MDR bacteria, phage therapy was not successful until the 1980s due to a lack of awareness and limited experimental data. In Georgia, phage therapy was used routinely with success; however, after the introduction of antibiotics and due to certain limitations associated with phage therapy, research in this field was brought to a standstill. Earlier studies conducted in Russia and Poland were uncontrolled and nonrandomized, and the clinical data were not sufficient to prove the effectiveness of the therapy (Żaczek et al. 2020). In the current scenario, however, numerous studies documenting successful bacteriophage therapy treatments have demonstrated that phage therapy can be developed into a novel treatment option (Kakasis and Panitsa 2019). One of the newer approaches to developing phage therapy is to construct phage libraries so that phages are readily available. Phages can be modified using CRISPR, increasing their specificity and uniqueness, which will enable them to selectively kill only antibiotic-resistant bacteria (Federici et al. 2021). Thus, companies are more likely to be able to obtain patents for unique phages and novel phage cocktails, which will help make phage therapy a commercially viable investment. This study aimed to systematically review and evaluate the current therapeutic rationale and clinical experience with phage therapy as a solution to treat infections caused by MDR bacteria.

## Phage therapy

**Fig. 1:**
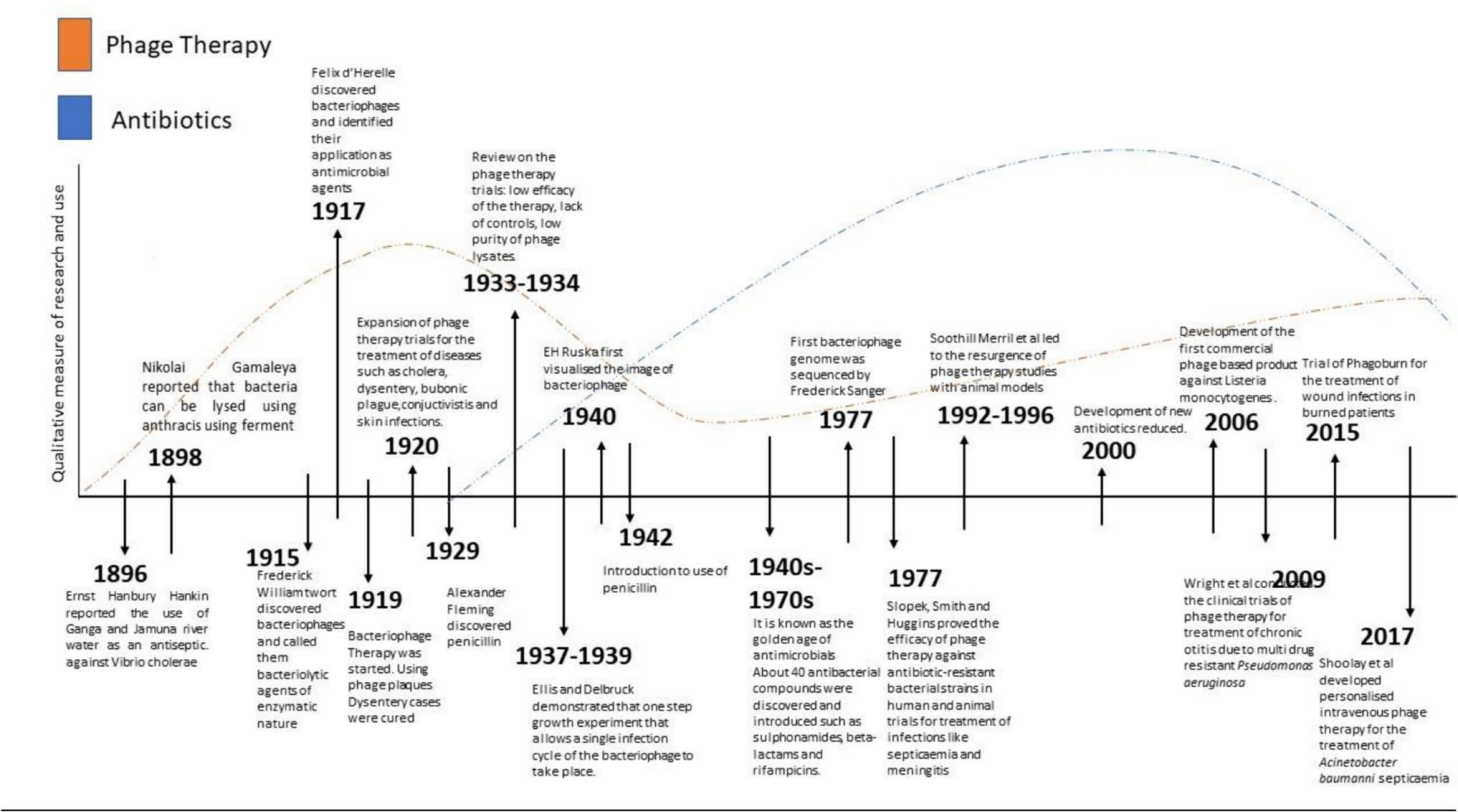
Timeline of the development of phage therapy and antibiotics. The graph shows the milestones and key events throughout history that led to the development of both phages and antibiotics. The figure is reproduced with permission from (Gordillo et al. 2019)

**Fig. 2:**
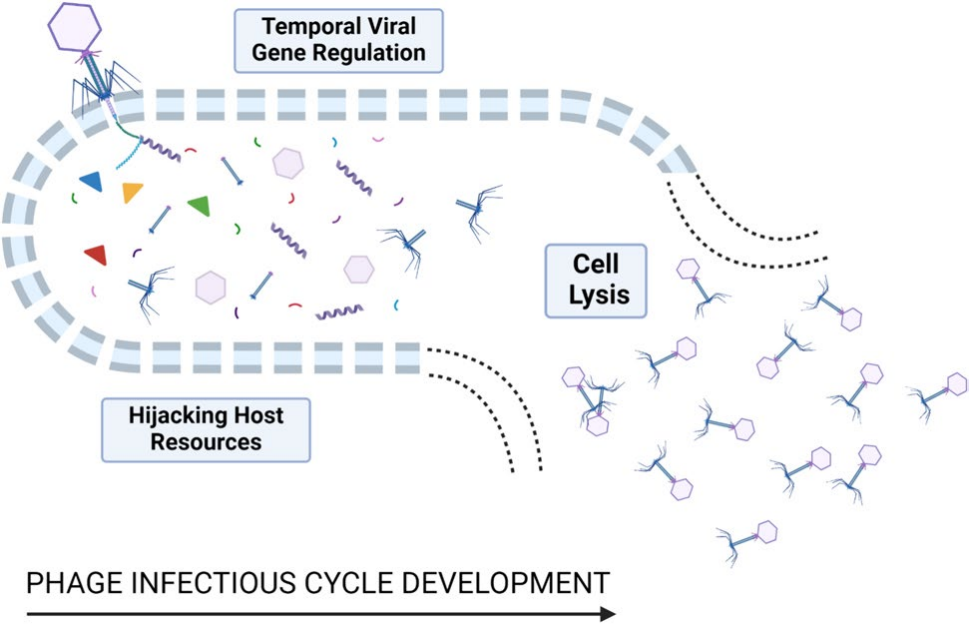
The bacteriolytic life cycle of phages**.** The figure is modified with permission from (Roach et al. 2017)

Bacteriophages/phages are viruses that infect bacteria; they are known to carry out reproduction in the host organism by using the host machinery since they lack the machinery to produce the energy and ribosomes that are required to produce proteins. According to their mode of action, after they enter bacterial cells, bacteriophages can be classified as virulent/lytic or temperate/lysogenic phages (Jamal et al. 2019). After entering target bacterial cells, bacteriophages can exhibit different life cycles: the lytic cycle, lysogenic cycle, and chronic infection. To study the therapeutic effect of phages, lytic phages are most considered. Ninety-six percent of the described lytic phages belong to the Caudovirales order, which consists of three families, namely, Myoviridae, Podoviridae, and Siphoviridae. The members of the family Myoviridae have notable features, such as a contractile tail and the largest capsid head, while the members of Podoviridae have a small capsid head and a short tail (Jamal et al. 2019). The family Siphoviridae is characterized by members with a small capsid head and a long tail. Bacteriophages replicate in cycles. Virulent or obligate lytic bacteria infect and cause lysis of the bacterial cell, thus killing it. After entering the host cell, temperate or lysogenic phages may integrate into the bacterial host cell or start the lytic life cycle. Temperate phages express viral genes that can change the bacterial phenotype, and this process is known as lysogenic conversion. Due to this process, bacteria can acquire pathogenic traits and antibiotic resistance determinants. Virulence phages are known to lead to lytic life cells once they infect a bacterial cell. Bacteriophages attach themselves to the surface receptors present on the surface of bacteria. This process is highly specific due to the selective binding between complementary receptors that are present on both the host cell and the bacteriophage. These receptors can be located on bacterial cell walls, appendages such as pili and flagella, or polysaccharide capsules (Stone et al. 2019). The phage injects its DNA into the bacterial cell after peptidoglycan degradation and pore formation. The majority of human pathogen-associated lytic phages belong to the family Microviridae of the order Petitvirales, which is also classified under Caudovirales. Lytic phages can have either single-stranded DNA or double-stranded DNA. The synthesis of new phage components takes place by redirecting the host metabolism to produce new phages by replication. The expression of phage genes occurs, and these genes cause the host synthesis machinery to reproduce phage components. Phage components are assembled to produce complete viral particles, and the individual viral components are assembled. Once the phage particles can enter the host cell, they replicate to produce fully formed phages, as depicted in Figs. 1, 2. Phage proteins such as lysins and holins cause the host cell to burst and release virions (Yang et al. 2014). The phage life cycles, i.e., the lytic and lysogenic life cycles. Lytic phages cause hydrolysis of the peptidoglycan layer, thus killing host bacteria (Malik et al. 2017). The progeny phages from the host cell are released, and the life cycle can continue as shown in Fig. 2. Temperate phages complete either the lytic cycle or the lysogenic cycle. In the lysogenic cycle, the phage genome is integrated into the host genome, and the resulting DNA is known as a prophage that can be induced to start the lytic cycle.

Phage therapy harnesses the lytic potential of bacteriophages to kill bacteria. Virulent phages, which consist of DNA/RNA enclosed in a capsid protein, are directly administered to patients (Saha and Mukherjee 2019). During the 1940s, in Western countries, the concept of phage therapy did not gain much interest due to the emergence of antibiotics and the ease of their application. However, in the erstwhile Soviet Union, phage therapy was used and is still in use (Sulakvelidze and Morris 2001). The Eliava Institute in Tbilisi, Georgia, is a leading research institute where extensive research on bacteriophages is being conducted (Kuntová et al. 2023). A part of the reason for the lack of interest in phage therapy was the inaccuracy and unreliability of phage trials. The decline in the use of phage therapy was also due to inadequate knowledge of bacteriophages, their mechanisms of action, and their biology. As therapeutic agents, bacteriophages can be problematic due to their narrow host range and the possibility of being destroyed by antibodies. The principal advantage of phage therapy is the specificity of bacteriophages toward the target bacteria. Bacteriophages are unique because they are self-limiting, causing few or no side effects (Lin et al. 1857). Additionally, in phage therapy, if bacteria become resistant to phages, then the phages can also evolve and thus reduce the chance of bacteria developing resistance against bacteriophages.

## Types of phage therapy

**Fig. 3:**
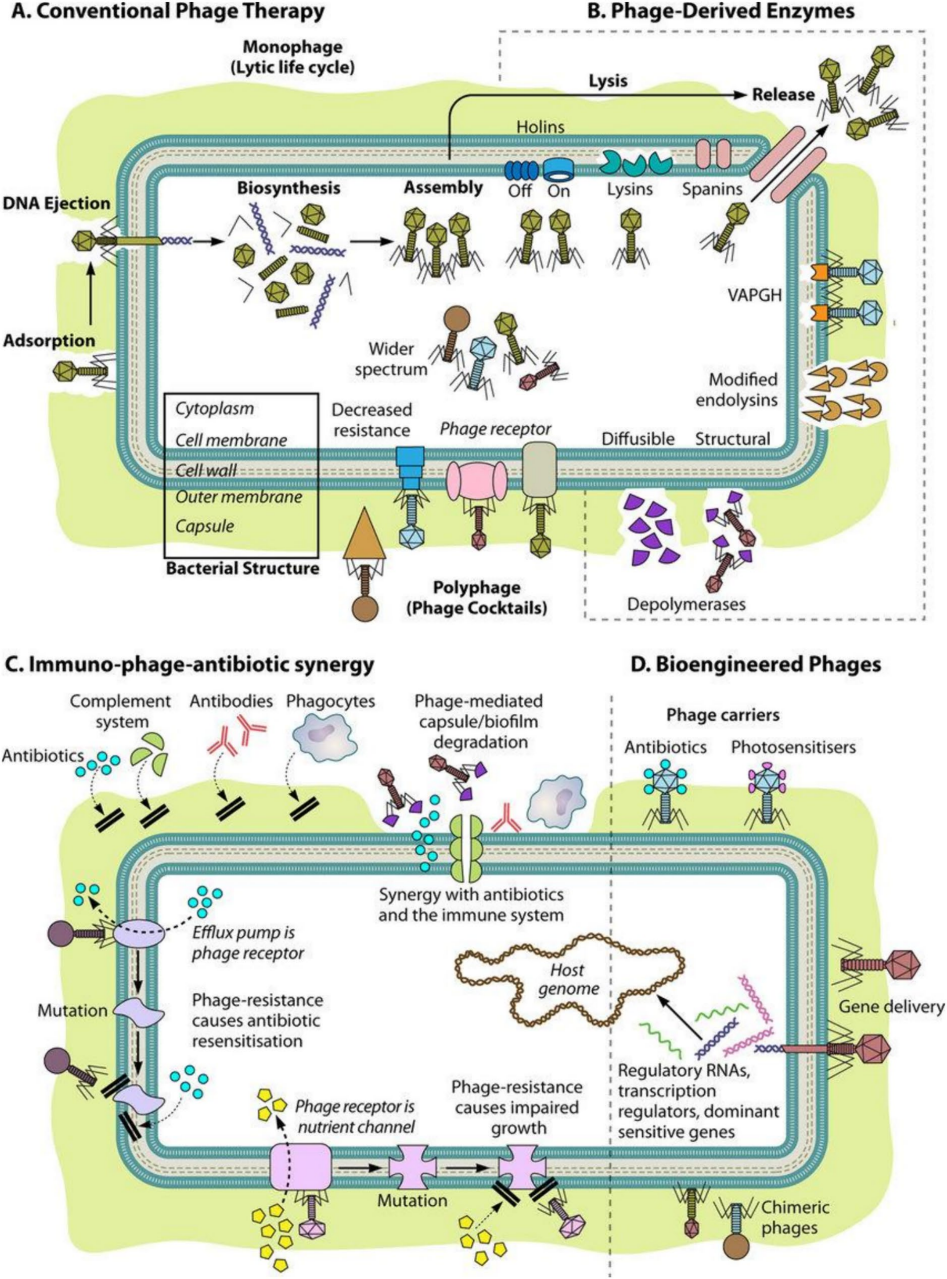
Different approaches for the delivery of phage therapy to bacterial cells. The figure is reproduced with permission from (Gordillo et al. 2019)

**Fig. 4:**
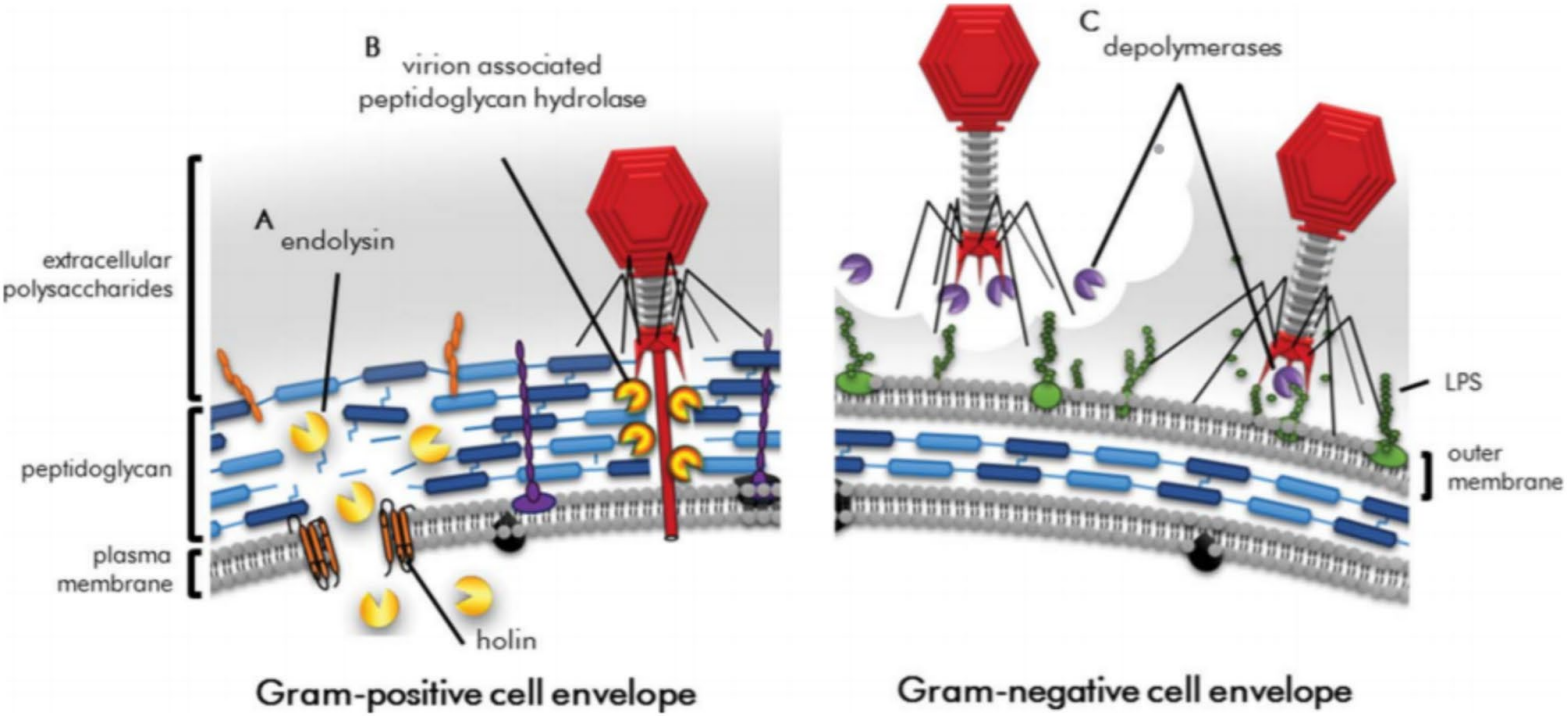
Diagram illustrating gram-positive bacterial cell lysis by exogenously applied endolysins. Endolysins cleave bonds in peptidoglycan and lead to lysis. Holins and endolysins cause cell lysis. The figure is reproduced with permission from (Roach et al. 2015)

There are different approaches to phage therapy depending on the mode of action of the bacteriophages. Phage therapy can be broadly categorized into five types, namely, conventional therapy, modified phage therapy, therapy with enzymes derived from phages, therapy with proteins derived from phages, and combination therapy. The different approaches to phage therapy delivery are depicted in Figs. 3, 4.

### Conventional phage therapy

Conventionally, phage therapy in humans is administered directly to patients by using virulent phages that are naturally isolated from the environment. In conventional phage therapy, phages are the only therapeutic agents administered to patients against bacterial infection, with the main aim being to cause lysis of the bacterial pathogen that is responsible for acute or chronic infection. For this therapy, lytic phages are preferred over temperate phages for the transfer of virulence genes into the genome of host bacterial cells (Bhargava et al. 2021). Monophage therapy uses a single phage preparation for the treatment of infections caused by MDR bacteria, such as vancomycin-resistant *E. faecium septicemia*. Clinical studies have shown that monophage therapy is effective for the treatment of urinary tract infections caused by *Enterococcus* bacteria (Zalewska-Piątek 2023). However, in monophage therapy, there are greater chances of the development of bacteria that evolve and become resistant to this therapy. Additionally, it is highly specific for each type of pathogen and requires matching between the phage and the pathogen. Thus, prophage therapy is considered a good alternative. In prophage therapy, a cocktail or combination of phages is designed such that multiple strains of bacteria can be targeted. Pyophage (PYO) and Intestiphage are two widely used generic phage cocktails. Phages that contain PYO specifically target *E. coli*, *S. aureus*, *P. aeruginosa*, and *S. pyogenes*. The intestine can target approximately 23 different enteric bacterial species. This combination of phages is used for the treatment of inflammation (Hibstu et al. 2022). Clinical studies have reported that the topical use of a cocktail of three lytic phages against burn-causing bacterial species, *P. aeruginosa* and *S. aureus*, is an effective treatment strategy with no side effects (Taati Moghadam et al. 2020). The limitations of this alternative are that it is time-consuming, and complex preparation steps are needed. Additionally, the chances of eliciting an immune response in patients are also greater. Human trials have shown that infections that cannot be treated with common antibiotics can be treated with phage therapy. Some of the issues that have to be addressed before this therapy can become more widespread include overcoming systemic side effects, phage resistance, narrow host range of phages, reduced immune system responses to phages, phage delivery issues, and challenges in phage manufacturing. Tables 1, 2 compares between phage therapy and antibiotics.

**Table 1:** Different routes of administration for phage therapy

Mode of administration	Advantages	Disadvantages	Solutions	References
Intraperitoneal	Diffusion to other sites. Effective delivery of phages	Less information about the diffusion of the bacteriophages to other sites	More than one delivery site	Qadir et al. (2018)
Intramuscular	Effective delivery of phages at the required target site	Lower volumes of bacteriophages can be administered and thus slower diffusion of bacteriophages to the target site takes place	Multidose course is needed	Kortright et al. (2019)
Subcutaneous	Localized and systemic diffusion	A lesser volume of phages can be delivered	Multidose course is needed	Steele et al. (2020)
Intravenous	Rapid diffusion to all parts of the body	Due to immune system reactions, rapid clearing takes place	Selection of low-immunogenic phages	Luong et al. (2020a), 2020b
Topical	At the infection site, a high quantity of phages can be delivered directly	Nonspecific site delivery	Easier mode of administration as the phages can be incorporated into a formulation like gels and dressings	Duplessis and Biswas (2020)
Oral	Easier form of delivery. Effective delivery of phages	Due to stomach acid, the number of phages decreases Nonspecific target site delivery of phages	Microencapsulation is required to safely deliver phages to the target area	Dąbrowska and Abedon (2019)
Aerosol	Easier form of delivery	Due to mucus and biofilms, phage numbers are reduced	Depolymerase enzymes can be used to reduce mucus	Prazak et al. (2021)

**Table 2:** Comparison between phage therapy and antibiotics (Kortright 2019)

Sr. No.	Parameters	Phage therapy	Antibiotics
1.	Bactericidal agent	Virulent phages cause cell lysis, which is bactericidal while bioengineered phages are bacteriostatic and prevent the growth of phages	Antibiotics cause cell death also known as bactericidal or can prevent the growth of bacteria known as bacteriostatic
2.	Discovery	Phage discovery is easy and rapid	Antibiotic discovery is time-consuming, expensive which requires drug design and development processes along with toxic potential testing
3.	Specificity	Bacteriophages are known to be highly specific and have a narrow spectrum of activity	Antibiotics have a broad spectrum of activity
4.	Microbiota disruption	Host specificity of phages leads to no disruption of the normal microbiota	Antibiotics with a broad-spectrum mode of action lead to disruption of the normal microbiota
5.	Side effects	Unlike antibiotics, phage therapy does not cause anaphylaxis in humans	Many include allergies and anaphylactic disorders
6.	Toxicity	Phages are nontoxic but lysed cell remnants may cause allergies in some cases	Toxicity levels can vary depending on the dosage and conditions of the patient
7.	Versatility	Phages are versatile, i.e., they display genetic diversity and abundance Phages can be delivered by various delivery systems and approaches Phages can be customized, i.e., phage cocktails can be administered depending on the bacteria that is being targeted	Antibiotics are not as versatile as compared to phages; to make them more effective, they can be used in combination with phages
8.	Resistance	The narrow range of activity of phages makes it specific; if resistance emerges then only a selected few bacterial species populations will be affected To reduce the chances of the development of resistance cocktail formulations can be used to treat antibiotic-resistant pathogens	A broad range of activity means that a large population of bacteria can be exposed to the antibiotic and there are greater chances of development of resistance against the bacteria and the potential for widespread resistance to emerge is greater
9.	Dosage	Dose concentration increases due to the process of host-dependent replication that increases the number of bacteriophages	The dose is dependent on the adsorption, distribution, metabolism, and excretion of the antibiotic
10.	Self-limitation	Once the target bacteria are killed, the bacteriophages stop functioning	No self-limitation occurs in antibiotics
11.	Availability of clinical evidence	Fewer clinical trials and studies are available; information about clinical relevance is still limited	Enough clinical studies and information about clinical relevance are available
12.	Development costs	Rapid and low-cost	Time-consuming and expensive
13.	Kinetics	Single hit or self-amplifying	Single hit
14.	Formulation	Fixed or variable	Fixed
15.	Immunogenicity	Low/yet to be established	Variable
16.	Regulation	Underway	Well-established

### Modified phage therapy

To overcome the challenges posed by conventional phage therapy, modifications of bacteriophages were considered and proven to be a successful alternative. To increase the overall efficacy of phages by delaying the immune response produced by the host’s bacteria, chemical PEGylation of phages was performed. Chemical PEGylation is the process of attaching monomethoxy-polyethylene glycol, a non-immunogenic substance, to the surface of a phage. Chemical PEGylation treatment of phages increases the circulation time of phages in the host due to evasion of T cells mediated by the immune response of the host (Karn et al. 2023). Another study showed that a single amino acid substitution in the lambda phage capsid (E) leads to a 1000-fold increase in circulation time, which is even more efficient (Vitiello et al. 2005). Virulent phages have been genetically engineered so that they can have a broad host range. A combination of two genetically engineered long tail fiber genes of phage T2 along with the broad host range of phage IP0008 produces a strong lytic phage (Levrier et al. 2024). Bacteria containing biofilms can be degraded by the enzyme disperin B; bioengineered *E. coli* phage T7 can be made to produce this enzyme, thus killing the target bacteria (Roy et al. 2018 Jan 1). Conventional treatments involve the direct killing of bacterial pathogens by phages; however, this can lead to the release of toxic substances such as endotoxins. Thus, lysis-deficient phages are being developed for use against infectious bacterial pathogens such as *S. aureus*, *P. aeruginosa*, and *E. coli*. Lysis-deficient phages have proven to be effective, as the levels of released endotoxins were reduced, and thus, a reduced immune response was noted (Egido et al. 2023). Lysis-deficient phages have been engineered such that the endolysin gene is inactivated; this gene encodes peptidoglycan hydrolase, which is required for bacterial cell hydrolysis. Another method of nonlytic cell death using phages is the delivery of lethal protein-encoding genes into bacteria. These genes are delivered to target bacteria by engineered filamentous phages. Lethal genes encoding holins, restriction endonuclease enzymes, modified lethal catabolite activator proteins, and addiction toxins are thus delivered into bacteria, causing programmed cell death (PCD) once the genes are expressed. One of the limitations faced while using filamentous phage therapy is that these phages are nonreplicating in nature, and there are chances that resistance can occur via the deletion of pili or alterations in bacterial cells (Hibstu et al. 2022). Resistance patterns similar to those found against antibiotics may develop in host cells against filamentous phages. To decrease the probability of host cells developing resistance against phages, engineered filamentous phages must target bacterial virulence factors, as these are less likely to be altered. This in turn helps decrease the chances of developing phage-resistant mutants (Zou et al. 2022). Phages can also be used to deliver photosensitizers, i.e., light-activated molecules that generate reactive oxygen species (ROS) that damage the cell structure and lead to cell death. This in combination with subsequent radiation from red light led to a greater number of *S. aureus* being killed, including MRSA strains of *S. aureus*. In this method, the phage is not injected into the bacteria but instead binds to the bacteria. This method causes minimal side effects when used on human epithelial cells (Correia et al. 2021).

### Therapy with enzymes derived from phages

**Fig. 5:**
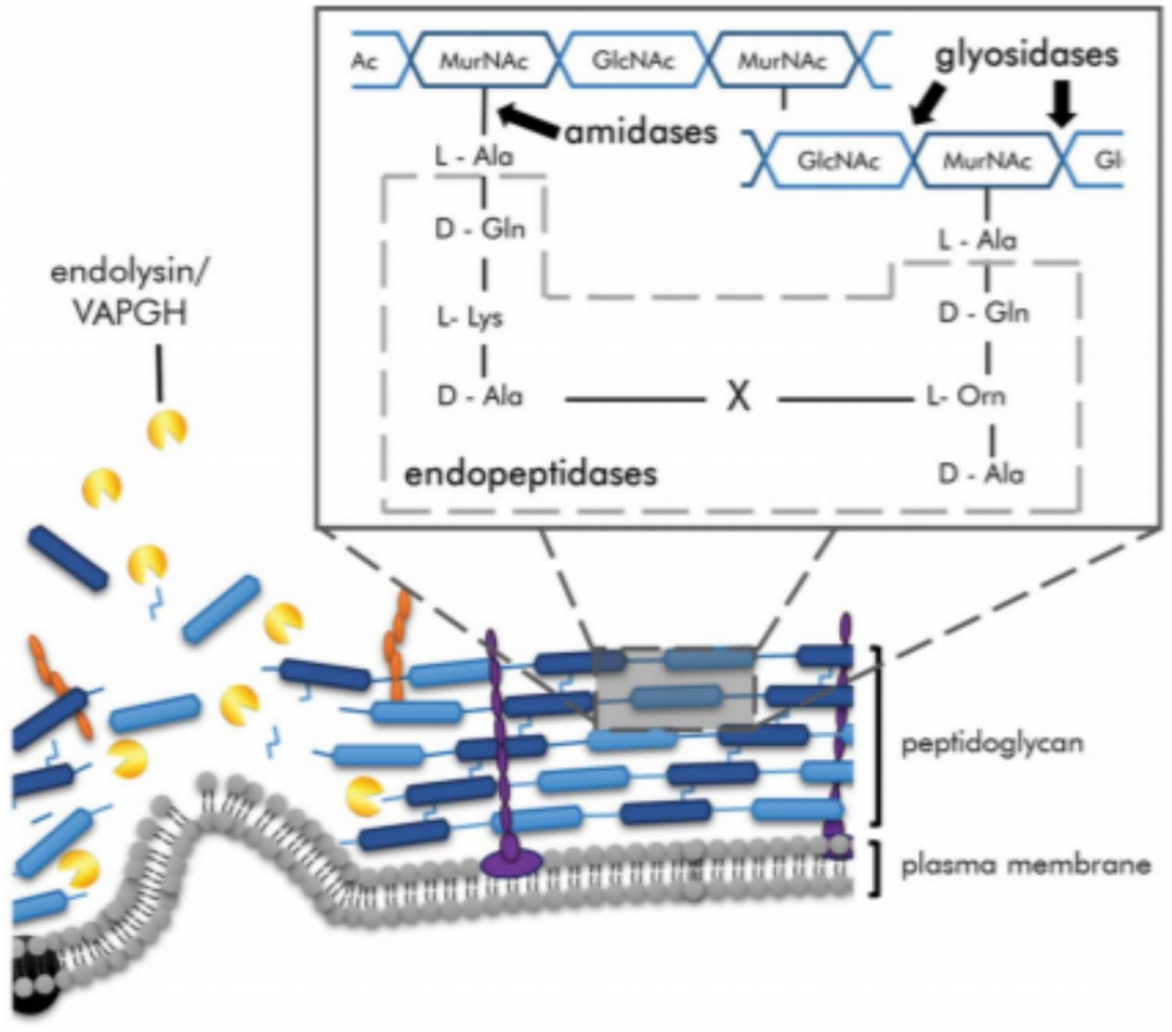
Diagram illustrating bacterial cells being attacked by phage-encoded proteins during lytic replication. Virion-associated peptidoglycan hydrolases are used by phages to puncture through the bacterial cell envelope. Depolymerases degrade polysaccharides that act as barriers to viral cell replication. The figure is reproduced with permission from (Roach et al. 2015)

Single phage-encoded enzymes provide an alternative to using entire natural or modified phages. Endolysins are enzymes that cleave covalent bonds in peptidoglycan and degrade the peptidoglycan cell wall of bacteria, leading to its death, as depicted in Fig. 5. Endolysins are specific for gram-positive bacteria and produce fewer side effects than antibiotics. These enzymes are classified into three major classes based on the peptidoglycan structure on which they act: amidases, glycosidases, and endopeptidases. Endolysins are effective against *Enterococcus faecalis* and *E. faecium*. According to clinical studies conducted on mice, subcutaneous injections of phage endolysins are effective at killing multidrug-resistant *S. aureus* infections in mice (Oliveira et al. 2018). The endolysin PlySs2, an endolysin derived from *Streptococcus*-infecting phages, is highly effective against MRSA, *Staphylococcus epidermis*, *Streptococcus pyogenes*, *Streptococcus pneumonia*, and *Streptococcus sanguinis*. Single endolysins are also effective against a combination of bacteria such as MRSA and *S. pyogenes,* as shown in mouse studies (Vander Elst et al. 2020). Since Gram-negative bacteria have an outer layer that protects the peptidoglycan layer from the action of endolysins, endolysins are not effective against gram-negative bacteria because they cannot penetrate the outer layer. An exception to this is the endolysin LysAB2 derived from the *A. baumannii* phage, which, due to its increased permeability, is effective against both Gram-positive and Gram-negative bacteria, along with MDR *Staphylococcus aureus* and *E. coli*. To be effective against Gram-negative bacteria, chemicals such as polymyxins and aminoglycosides that permeate the bacterial cell, as well as chelating agents such as EDTA, are used so that endolysins can act on the bacteria after the outer membrane is permeabilized (Sun et al. 2022). Modified endolysins can also be used in this process. Artilysin is an example of a bioengineered endolysin; polycationic oligopeptides are added to the structure of the endolysin, allowing it to permeabilize the outer membrane of the bacteria, thus making it effective at killing Gram-negative MDR bacteria such as *P. aeruginosa* and *A. baumannii* (Rahman et al. 2021). Endolysin therapy against Gram-positive pathogens has many advantages; unlike phage therapy, endolysins do not involve a lag phase and are thus effective immediately. Additionally, the development of resistance toward endolysins has not been observed thus far. Holins act by forming pores in bacteria in combination with endolysins. Endolysins pass through these pores formed by holins and move from the cytoplasm into the periplasmic space. Endolysins are thus able to interact with peptidoglycan and ultimately cause lysis of bacterial cells (Rahman et al. 2021).

### Therapy with proteins derived from phages

Virion-associated peptidoglycan hydrolases (VAPGHs) and enzyme depolymerases lead to the degradation of the bacterial cell wall. VAGPH is present on the phase base plate and degrades the peptidoglycan layer of the bacterial cell so that the phage can inject its genomic material into the pathogen cell (Rodríguez-Rubio et al. 2013). Depolymerases are phage-encoded enzymes that target the bacterial capsule, which consists of peptidoglycan. Once peptidoglycan is degraded, depolymerases can act on secondary host receptors located on the cell wall, as depicted in Fig. 4. The depolymerases and virion-associated peptidoglycan hydrolases can degrade biofilms that are produced by microorganisms. By acting on capsules, which are the virulence determinants of many bacteria, such as *Haemophilus influenzae*, *E. coli*, and *Streptococcus pneumoniae*, depolymerases allow the penetration of antibiotics to kill bacteria once the defense mechanism is lost. The polysaccharide-degrading action of depolymerase allows antibiotics to penetrate and eradicate biofilms formed by *K. pneumoniae* (Knecht et al. 2020).

### Combination therapy

Combination therapy using phages and antibiotics produces an increased synergistic effect. It leads to an increased antibacterial effect, and the development of resistance is very limited. Antibiotics are carried into the target cell using phages; antibiotics can be linked to surface receptors or may be integrated within the phage itself. Antibiotics such as chloramphenicol can be linked to the phage protein coat by an aminoglycoside bond. This method is more effective than the use of drugs. An advantage of this method is that already approved antibiotics can be used, and there is no need to design new antibiotics. To enhance the synergistic effect, combination therapy was modified such that nonlytic filamentous phages can be used as adjuvants (Li et al. 2021). Thus, bacteria such as *E. coli* become more susceptible to therapy due to modifications in the structure inside the cell, which increases the effect of the drug. Phage-Antibiotic Synergy (PAS) combines these two approaches. Comeau et al. reported that certain antibiotics at sublethal concentrations can cause *E. coli* to produce virulent phages; this combination is only effective for certain combinations of antibiotics and bacteria. PAS is effective against *E. coli* due to the use of antibiotics such as B lactam, quinolones, and mitomycin C, and for *P. aeruginosa*, antibiotics such as chloramphenicol, carbenicillin, and tetracycline are useful (Li et al. 2021). One of the disadvantages of PAS is that sublethal concentrations of antibiotics may lead to resistance development in bacteria other than the target cells.

## Bacteriophage resistance mechanisms

**Fig. 6:**
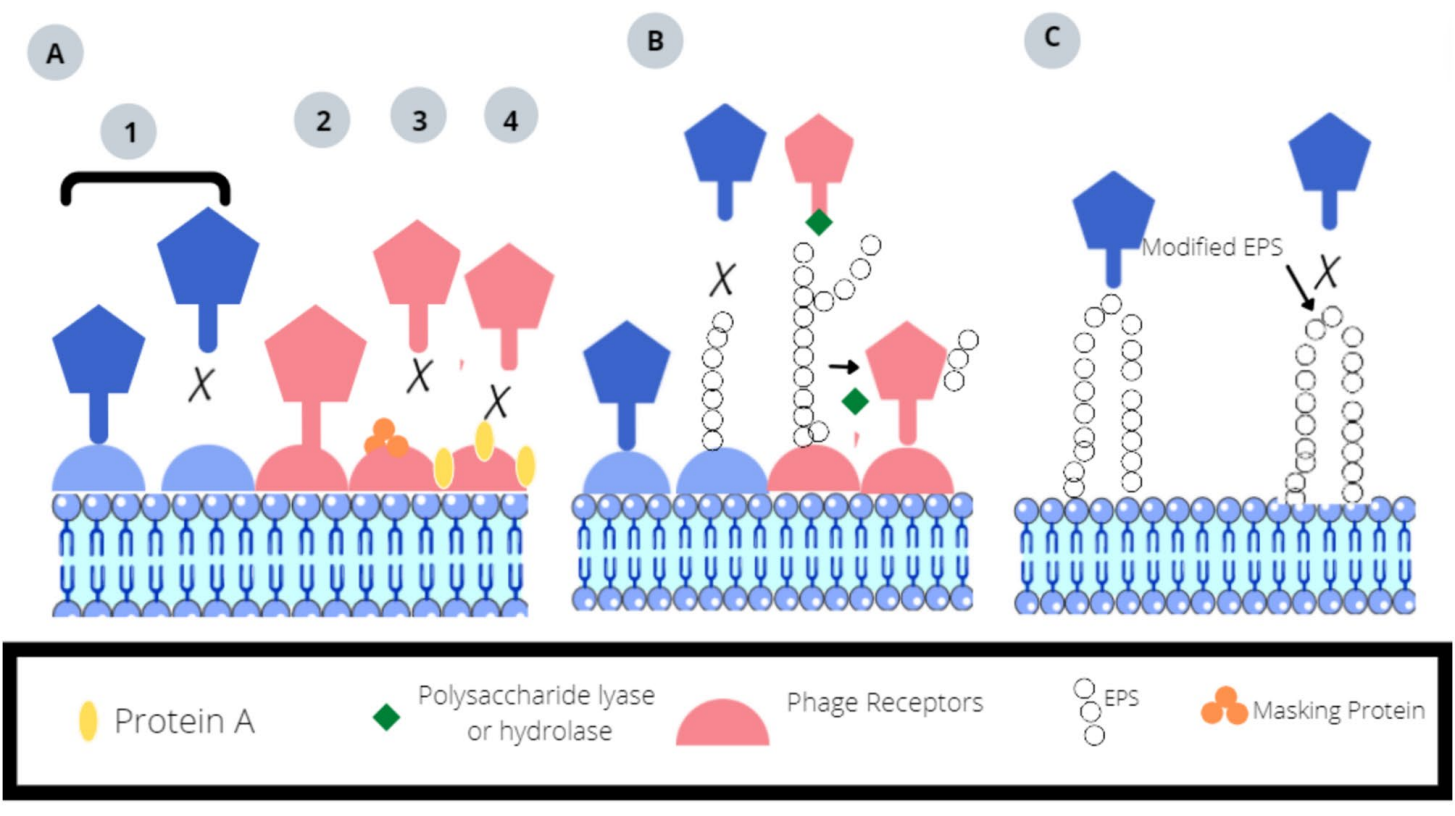
Different strategies used by bacteria to block phage adsorption. The figure is reproduced with permission from (Labrie et al. 2010). **A** At the first stage of adsorption, bacteriophages require specific receptors on bacteria for recognition and attachment. Step 1: Bacteria modify their cell surface receptors to become resistant to phage. Phages adapt and recognize these new receptors. Step 2: Bacteria also produce certain proteins that mask their cell surface receptors. Steps 3 and 4: For example, *Staphylococcus aureus* produces protein A, which reduces cell surface adsorption. **B** Phage adsorption can be blocked by the production of exopolysaccharides (EPS). To overcome this, phages can produce polysaccharide lyases or hydrolases to cleave EPS. **C** Phages have evolved to recognize polysaccharides such as the antigens O and K

Like antibiotics, phage therapy is also prone to bacterial resistance. Clinical studies have shown that 50% of sepsis infections are caused by phage-resistant bacteria. Resistance to phages by bacteria can evolve through several mechanisms. One mechanism is spontaneous resistance to phages. For a bacteriophage to infect a bacterium, surface receptors are needed. In bacteria that are resistant to phages, their surface receptors may not be expressed or may be mutated, thus reducing the chances of phage adsorption onto the surface of the bacteria and making the phages ineffective against the bacteria. This mechanism of resistance employed by bacteria is effective against both phages and antibiotics. Phages can adapt to recognize new receptors. Phage absorption can be blocked by the production of exopolysaccharides. To cleave exopolysaccharides, phages produce polysaccharide hydrolase or lyase enzymes. Another mechanism is acquired resistance, which can occur due to the transfer of genes that code for antibiotic resistance. These genes can be carried by accessory proteins such as plasmids and temperate phages and can be transferred (Labrie et al. 2010) between bacterial species. To overcome the evolution of phage-resistant bacteria, phage therapy treatments should use a cocktail of phages. As many phages are combined in a phage cocktail, the chances of developing resistance to phages decrease. Genetic engineering can help to make phages unique and decrease the chances of developing bacterial resistance. Figure 6 depicts the resistance mechanism of bacteriophages.

## Phage pharmacology

**Fig. 7:**
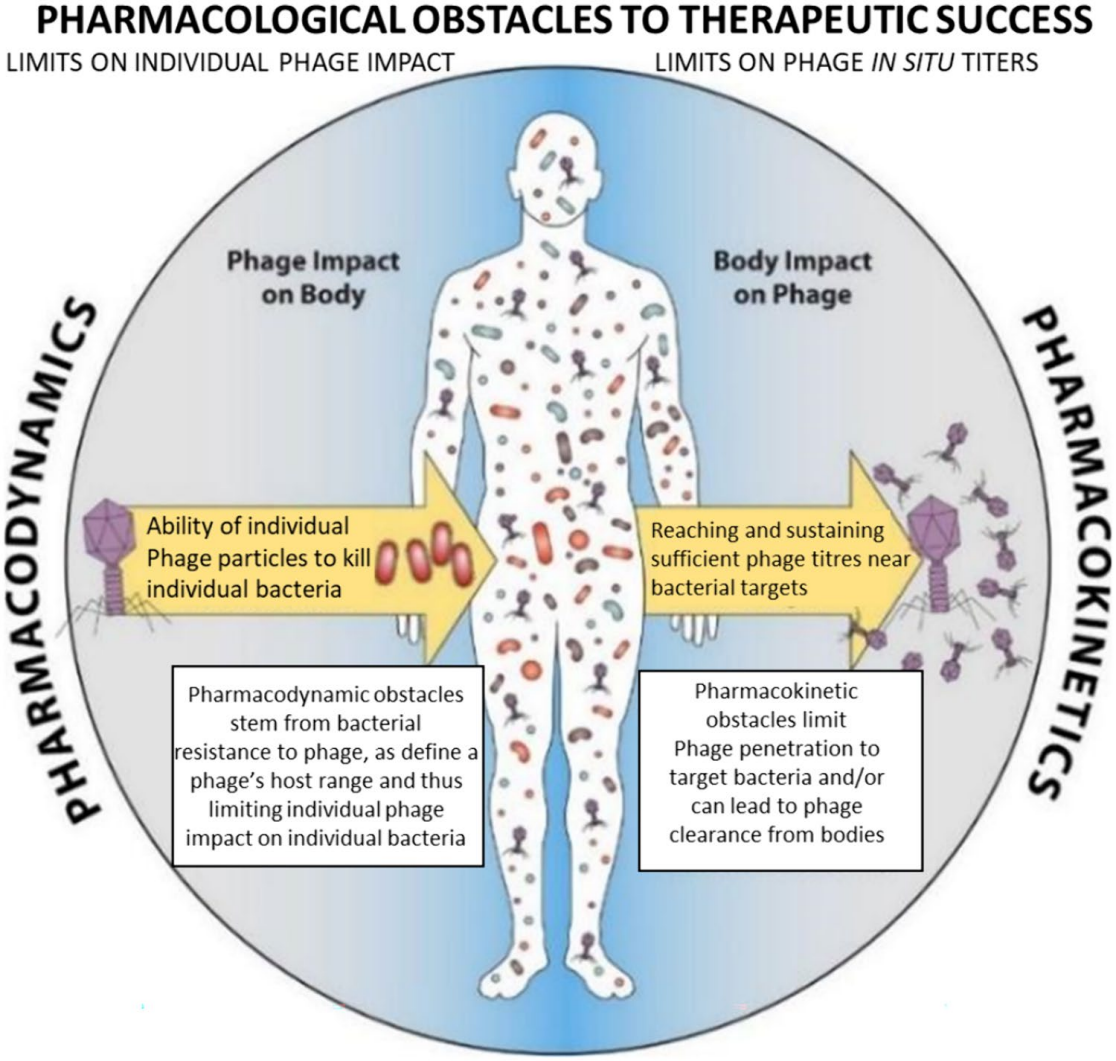
Pharmacodynamics explain the impact of the phage on the body, and the pharmacokinetics of the phage explain the impact of the body on the phage. The pharmacodynamics of phage therapy can explain the ability of individual phage particles to kill individual bacteria, while the pharmacokinetics of phage therapy can help us to understand the number of phage titers that are sufficient to effectively target bacteria. The figure is modified with permission from Dąbrowska and Abedon (2019)

Phage pharmacology is the in-depth study of interactions between bacteriophages and host cells (Stone et al. 2019). Pharmacodynamics and pharmacokinetics are the two components of phage pharmacology that are involved in drug action. The interaction of drugs with their specific receptors, which are linked to transduction systems that cause changes in the cells or body of the patient, is known as pharmacodynamics. There can be two types of impacts of drugs: positive impacts that maintain or restore the health of the individual and negative impacts that include toxic side effects. Pharmacokinetics is another component of phage pharmacology. Drug absorption and distribution are the movement of the drug in the body, first through blood and then into organs (Dąbrowska and Abedon 2019). Drug metabolism occurs when drugs are converted to their active form due to metabolism, and excretion of the drug is the removal of the drug from the body. The pharmacodynamics of phages help to determine their efficacy. Individual phage particles can kill individual bacteria. Bacteriophages are known as active antibacterial agents against pathogenic bacteria due to their low toxicity and self-amplification. The relatively low toxicity, action against biofilms and ability of phages to inhibit bacteria are some of the key features associated with phages. Bacteriophages do not disrupt the microflora of the body, and upon interaction with the body’s immune system and metabolism, phage virions can be degraded, but there is no production or accumulation of toxic byproducts (Danis-Wlodarczyk et al. 2021). Phage therapy is safe since intact phages cannot interact with the body’s metabolism by specific binding or any manipulation of the body’s tissues. The key features, such as specificity toward the target bacteria and their single-killing kinetics, contribute to the unique pharmacodynamics of phages (Zalewska-Piątek 2023). The ability of bacteriophages to kill bacteria can be measured by the killing titer, which is the phage density in a solution that is required to kill the bacteria. The pharmacodynamics and pharmacokinetics of phage therapy are depicted in Fig. 7.

**Fig. 8:**
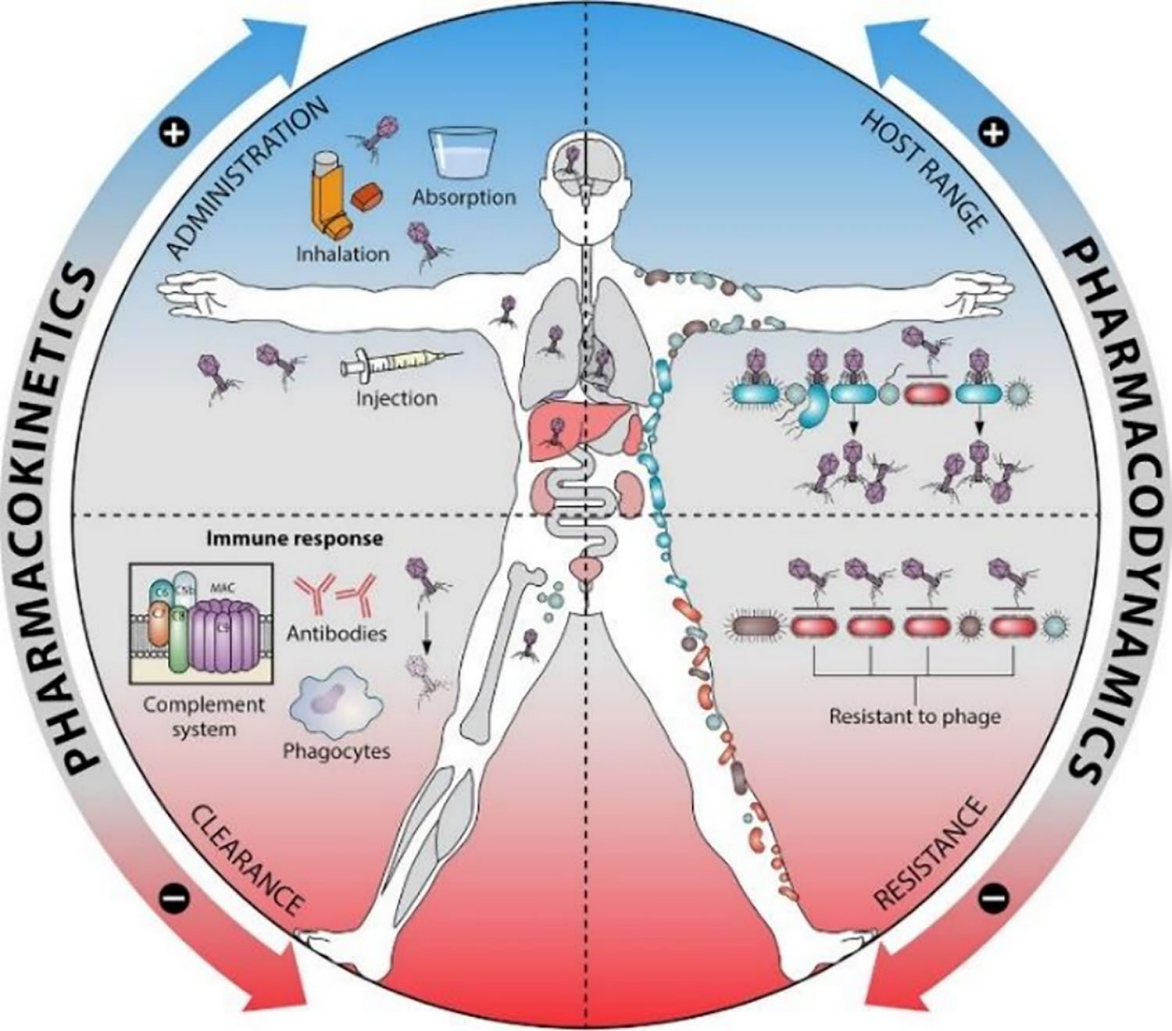
Factors affecting phage pharmacokinetics and pharmacodynamics. Factors such as phage administration and immune responses by host cells can affect the pharmacokinetics of phage therapy, while host range and resistance can affect the pharmacodynamics of the therapy. The figure is reproduced with permission from Dąbrowska and Abedon (2019)

Pharmacokinetics studies how an organism reacts to a drug. It can be defined as the body’s impact on the phage. The absorption of the drug depends on how the drug has been administered to the patient, i.e., by systemic delivery of the drug into the circulatory system of the body so that the entire body is affected or by nonsystemic delivery in which the drug is administered at a specific site to localize the effect of the drug to a specific site (Grogan and Pharmacokinetics 2023). The distribution and movement of phages in the body are dependent on phage dosage. Phage movement is directly proportional to the dosage of phages in the body. Metabolism occurs when phage particles become inactivated as they interact with the immune system. Excretion leads to the elimination of phage particles from the body. Obstacles in the pharmacokinetics of phage therapy can limit the absorption and distribution of phages or can lead to their elimination by metabolism and excretion. There are two mechanisms of action, namely, active treatment and passive treatment. In active treatment, once the phage has been injected into the host, reproduction and transmission of the phage take place, and by secondary infection, most of the bacteria are killed. In passive treatment, however, the initial dose of bacteria is sufficient to kill the bacteria by primary infection (). The factors affecting phage pharmacokinetics and pharmacodynamics are depicted in Fig. 8.

## Applications of phage therapy

Phage therapy has been used in several clinical trials for the treatment of several conditions. The treatment of skin ulcers by phage therapy has proven to be successful, which is highly useful since antibiotics are ineffective in the treatment of infected skin ulcerations in some cases. For the treatment of patients resistant to other treatments, a microbial wound healing polymeric biodegradable film that contains a bacteriophage cocktail is used. This wound-healing preparation is known as a phagobioderm. Clinical studies have shown that when the phagobioderm was used alone or in combination, the results were positive in approximately 70 out of 100 patients who were tested (Stacey et al. 2022). Wounds can be the result of surgery, accidents, or burns; phages are used for treating prophylaxis by preventing infections at the wound site. In Georgia, phages have been used to treat surgical infections and MDR infections. Phage preparations can be introduced into the wound by using bandages soaked in a liquid preparation or by spraying the wound with phage preparation after surgery. In 2000, the Phage Bioderm Company released a phage containing antimicrobial polymeric biocomposite materials. For deeper wounds, phagebioderm preparation along with pyophage was administered for wound treatment. In a clinical study, 107 people who were resistant to conventional antibiotic treatment for ulcers were treated with Phage Bioderm, which yielded 70% positive results (Markiewicz-Gospodarek et al. 2022). Burn wounds are highly susceptible to bacterial infections. Phage therapy against the bacterial species *P. aeruginosa, S. aureus, E. coli,* and *K. pneumoniae* has a 90% success rate. Among these, *P. aeruginosa* is known to be the most common cause of death in burn patients (Maslova et al. 2021). In clinical trials conducted in France and Belgium, PP1131, a phage cocktail of lytic anti-*Pseudomonas aeruginosa* bacteriophages, was shown to be effective in reducing the bacterial concentration in infections; it passed phase I clinical trials.

**Fig. 9:**
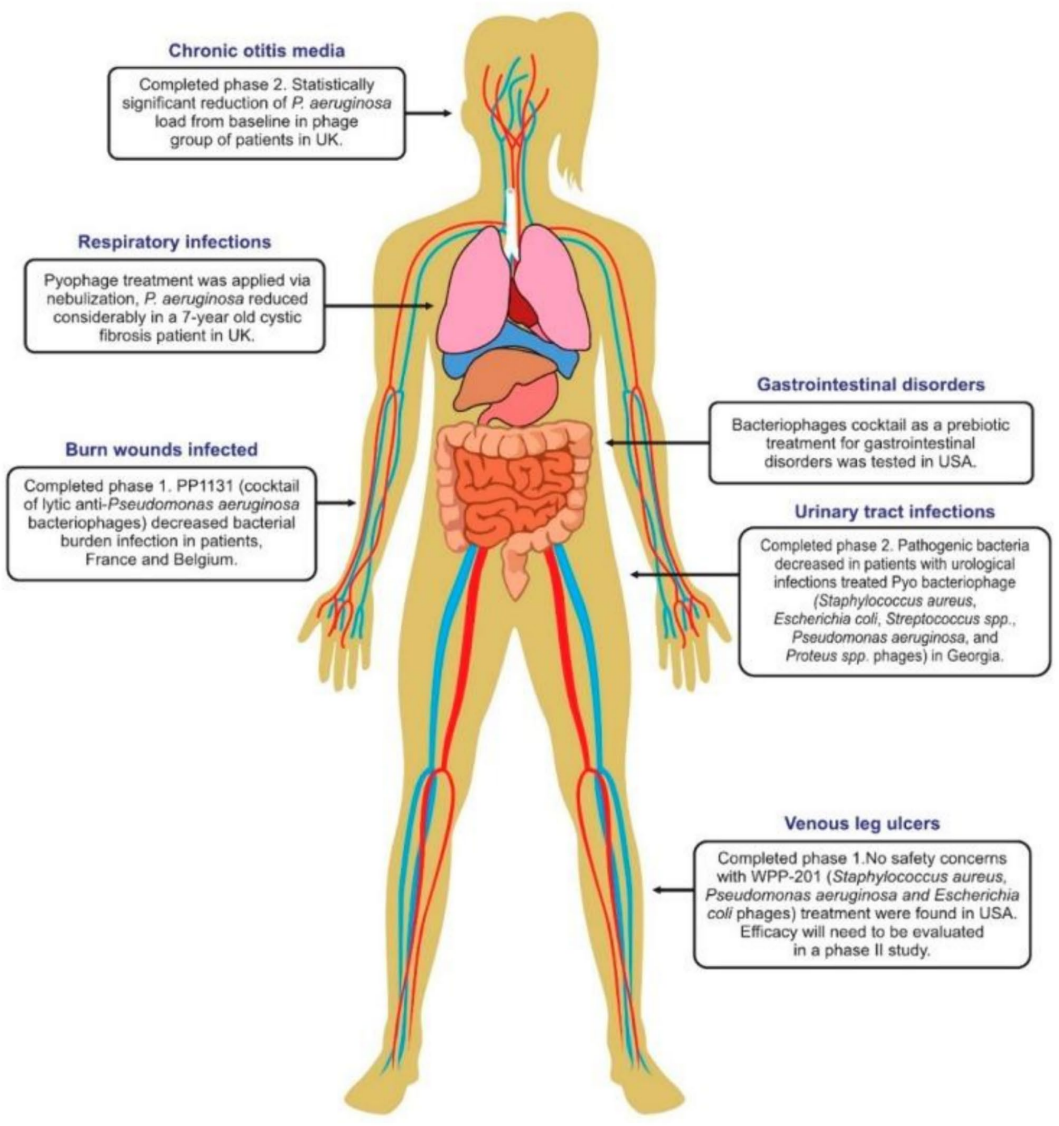
Human phage therapy trials and the range of target sites and infections. The figure is reproduced with permission from (Romero et al. 2019)

Phage therapy is also effective in the treatment of bacterial respiratory tract infections. In clinical studies, phage treatment has been successful in the treatment of patients with cystic fibrosis (CF). In clinical studies of CF patients, phage therapy against *P. aeruginosa* strains has shown a success rate of 86.4% (Chang et al. 2018). In a case study conducted in 2011 in Georgia involving a seven-year-old patient suffering from CF due to the bacteria *P. aeruginosa* and *S. aureus*, antibiotics were not effective against the bacteria. As an alternative, a phage cocktail consisting of a Pyophage cocktail along with Sb-1 was administered to the patient by nebulization at an interval of four to six weeks. This treatment was effective in reducing the concentrations of *P. aeruginosa* and *S. aureus*. This prophage cocktail is known to act against *E. coli*, *P. aeruginosa*, *Streptococcus*, and *Proteus*. The Sb-1 phage was active toward *S. aureus* and reduced its number, thus controlling the infection (Abedon et al. 2021). The use of spray-drying phages as a mode of delivery and the use of a cocktail of ten phages has helped improve the outcomes of phage therapy against bacterial infections. The use of phage therapy for the treatment of UTIs is highly effective. In clinical trials conducted in Georgia on patients suffering from urological infections, phage therapy has completed phase II clinical trials and is effective in reducing the concentration of bacteria such as *Staphylococcus aureus*, *E. coli*, *Streptococcus* sp., *Pseudomonas aeruginosa*, and *Proteus* spp. (Zalewska-Piątek and Piątek 2020). In a clinical trial on humans, phage preparations were used to treat 42 patients suffering from leg ulcers caused by the bacterial species *P. aeruginosa, S. aureus*, and *E. coli*. The trial was successful, and it passed phase I clinical trials. To test the efficacy of phage preparations, phase II clinical trials must be conducted (Chegini et al. 2020). To test the efficacy and efficiency of phage therapy, clinical trials were conducted for the treatment of chronic ear infections caused by the bacterium *P. aeruginosa*. Twenty-four patients suffering from ear infections were administered a phage cocktail composed of six phages produced by Biocontrol Limited. The treatment was successful, and no side effects were observed. Phage therapy has passed phase I and phase II clinical trials for this disease (Stacey et al. 2022). The results of human phage therapy trials and the range of target sites and infections are depicted in Fig. 9.

Biofilm-associated infections are difficult to diagnose and treat because bacteria can evade the immune system and antibiotics. Biofilm-mediated musculoskeletal infections can be treated with phages, especially if the hardware cannot be removed. The results of in vitro studies suggest that some phages are active against a variety of biofilms (Vidakovic et al. 2018) and with different extracellular matrix types. The use of phages in treating musculoskeletal infections was described in several case studies before the twenty-first century, primarily from Poland and Georgia. Phage treatment is generally considered successful in up to 90% of cases (Weber-Dabrowska et al. 2000). In recent years, several clinical phage treatments for musculoskeletal infections have been published. Periprosthetic joint infections, spinal infections, trauma-related infections, and craniectomy-related infections are among the musculoskeletal infections that phage therapy has been used to treat. Phage therapy has been administered both in vitro (Liu et al. 2021) and locally to treat musculoskeletal infections (Ferry et al. 2020). A case report by Onsea et al. described the use of phage therapy to treat four orthopedic infections with standardized treatment approaches using locally administered phages (Onsea et al. 2019). The phages were administered intraoperatively and postoperatively three times a day for 10 consecutive days. During phage therapy, all patients received antibiotics, and their clinical status was monitored daily. During the 8- to 16-month follow-up, no recurrence of infection was observed (Onsea et al. 2019).

## Benefits, drawbacks, and future prospects of phage therapy

Bacteriophages act as antibacterial agents, and lytic phages are highly effective against multidrug-resistant bacteria (Hibstu et al. 2022). Phages also display the property of autodosing, i.e., they can multiply and increase in number depending on the bacterial density against which they act. Thus, the dose or amount of phage population needed to kill bacteria can be controlled (Hyman 2019). Additionally, phages are not toxic since they consist of nucleic acids and proteins that are inherently non-toxic to the host. Pure phage preparations are only required for use when there is a chance of anaphylactic response to bacterial components such as endotoxins present in the phage preparation (Loc-Carrillo and Abedon 2011 Mar). Bacteriophages cause minimal disruption of the normal microflora of the host. As phages have a narrow spectrum of activity, the chance of harming natural microflora is less, and thus, there is no damage to the patient’s body (Batinovic et al. 2019). Bacteriophages use a mechanism of action different from that of antibiotics to infect and kill bacteria. Thus, phage therapy can be used to treat antibiotic-resistant infection-causing bacteria since antibiotic resistance does not mean that the bacteria are also resistant to phages (Hibstu et al. 2022). Bacteriophages have a narrower potential for inducing resistance as well as a narrower activity range and can kill bacterial strains that can evolve and acquire phage resistance mechanisms (Hibstu et al. 2022). Phages are versatile in their application forms and formulation development and can be combined with antibiotics. Phage cocktails can also be used to increase the spectrum of activity against bacteria (Abedon et al. 2019). Unlike antibiotics, bacteriophages are effective at degrading biofilms when combined with biofilm-degrading depolymerases (Chang et al. 2022). Bacteriophages have single-dose potential since phages can infect host cells and replicate in the host. Thus, only a single dose or a few doses of phages are needed. This increases the convenience of phage therapy compared to antibiotics (Hibstu et al. 2022). Additionally, since phages can achieve active therapy by replicating within the host and increasing their density according to the density of the infecting bacteria, only low doses of phage are required for treatment (Islam et al. 2023). These unique properties of phages make phage therapy an effective and viable alternative for the treatment of MDR bacterial infections.

However, despite its effectiveness, phage therapy also has some drawbacks. Bacteriophages have a narrow spectrum of activity and thus show selective toxicity toward specific bacterial strains. Therefore, phage treatment is specific for each bacterium, and treatment can only begin after the bacteria causing the infection in the patient have been identified, after which the phage is chosen to act against it. This also indicates that a mixture of many phages will be required to target several different bacteria (Jamal et al. 2019). Before the administration of phage therapy, in-depth knowledge of phage biology is needed. Bacteria can evolve and acquire resistance to phages; to overcome this challenge, bioengineered phages and combination therapy can be used. However, additional clinical studies are required because phage behavior inside the human body is not well characterized, increasing the risk of administration. Characterizing each strain used in a phage cocktail can be laborious and expensive (Gavric and Knezevic 2022). Different types of routes of administration for phage therapy are represented in tabular form in Table 1. Phage therapy is still in the early stages of development and faces challenges regarding the indirect effects it might have on human patients. Bacteriophages do not cause a direct effect on human cells but can produce byproducts such as toxins or viral products that are released when the bacterial cell bursts. This could lead to an immune response in the patient. Some bacteriophages can lead to the integration of antibiotic resistance genes into the viral genome, which will then be transferred to the bacterial species. Thus, it is crucial to ensure that the bacteriophage used in the treatment does not incorporate any antibiotic resistance genes into the bacterial genome (Podlacha et al. 2021). Under modern regulatory standards, obtaining approval for clinical trials is the greatest challenge for phage therapy.

**Fig. 10:**
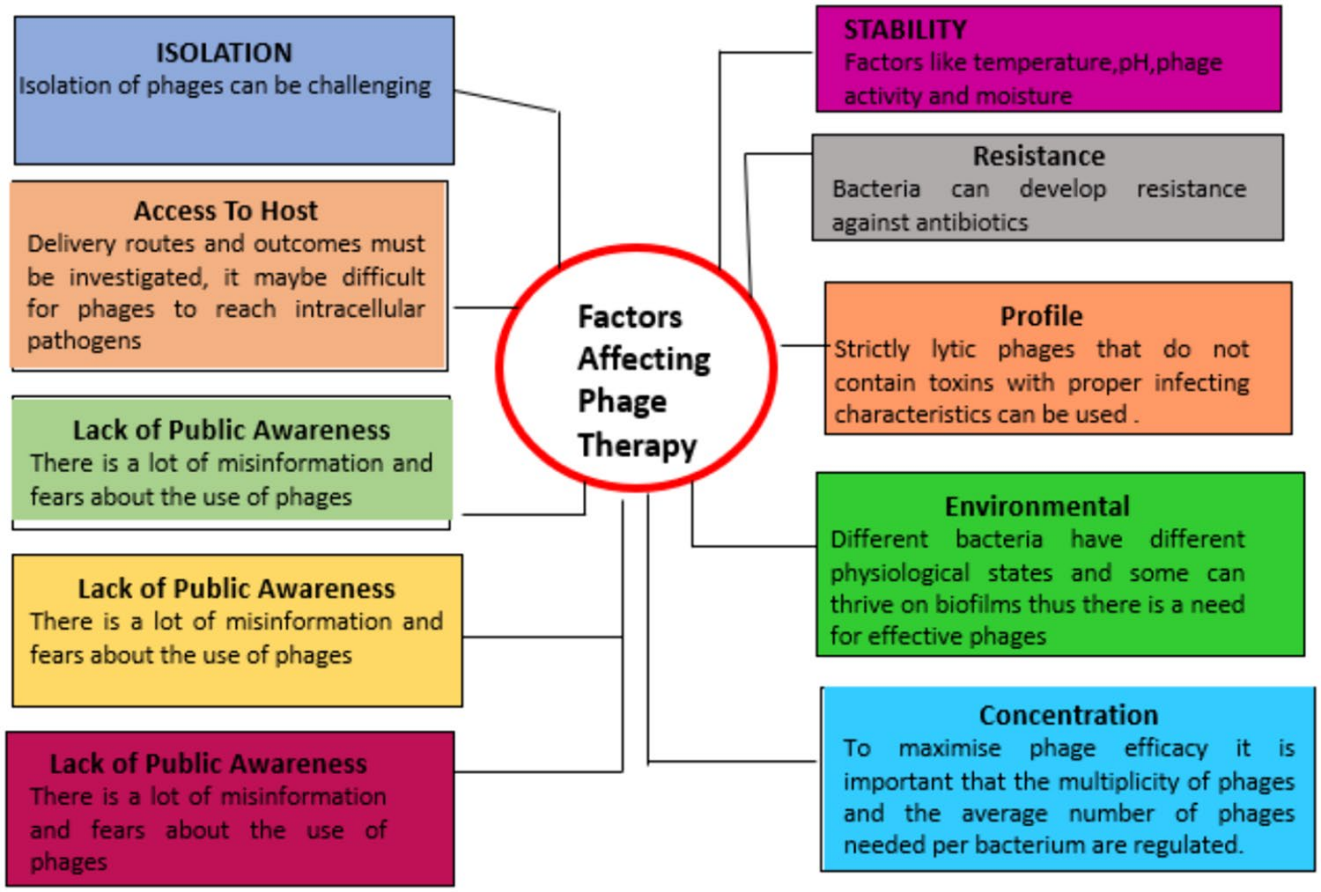
Intrinsic and extrinsic factors affecting phage therapy. The figure is reproduced with permission from Oliveira et al. (2018)

The establishment of the genetic makeup and stability of phages are two of the major hurdles to approval. This is because regulatory standards have been designed for chemical compounds, while phages, on the other hand, are natural biological organisms that can evolve with the bacterial host (Furfaro et al. 2018). Thus, it is difficult to assess phage therapy, especially phage cocktails, which are composed of multiple strains of phages. The specificity of phages is an important factor in determining their effectiveness. The specificity of phages means that there are no off-target effects. This also means that more time is required for the discovery of the specific phage after determining the strain of the bacterial pathogen. The intrinsic and extrinsic factors affecting phage therapy are depicted in Fig. 10. The renewed interest in phage therapy can be seen in the increasing number of phage products, clinical trials, and research being conducted (Gondil and Chhibber 2021). A large number of pharmaceutical companies are researching bacteriophages in clinical trials. An FDA-approved phage product named LMP-102 is being used in the poultry industry. It is a bacteriophage cocktail phage preparation of six phages and is effective against *Listeria monocytogenes,* which is a gram-positive bacterium. Companies such as Novolytics Limited, Phico Therapeutics, and Biophage Pharma are actively involved in developing phage products against *S. aureus* (Moradpour and Ghasemian 2020). These successful results in animal models have led to clinical trials and indicate that this therapy is a practical alternative to antibiotic treatment. However, more research and clinical data are required about phage pharmacology, the stability of phages and phage cocktails within delivery formulations, and the development, optimization, and characterization of novel formulations. Therefore, more clinical trials must be undertaken to facilitate the widespread clinical application of phage therapy. The companies involved in the development of phage-based products are listed in Tables 3,4.

**Table 3:** Companies involved in the development of phage‐based products

Company	Product	Target application	References
Biocontrol (UK)	Phage products are being developed to cure diseases such as otitis and lung infections	Clinical trials of phage products to cure infections caused by *Pseudomonas aeruginosa* have been completed	Moradpour and Ghasemian (2020)
Novolytics (UK)	Still in the development stage—gels for targeting *S. aureus*	A gel containing a cocktail of phages targeted against *S. aureus* to treat nasal infections	Gildea et al. (2021)
Bio phage Pharma (Canada)	Phage‐based products for a range of applications	A large bank of phages is being isolated from natural sources for use in phage therapy applications,	Moradpour and Ghasemian (2020)
Microphage	Screening test for methicillin-resistant *Staphylococcus aureus*	Used for the identification of methicillin-resistant *Staphylococcus aureus*	Dabbagh et al. (2019)
Biotech laboratories	FASTPlaque-response	Development of phage-based diagnostic tools that can be used for testing samples for the presence of Mycobacterium	Dabbagh et al. (2019)

**Table 4:** Various prospects of phage therapy research in the future

Future direction	Overview	Key focus areas	Expected outcome	Challenges	References
Phage selection and optimization	Determine and enhance efficient phage strains	Genetic engineering and high-throughput screening	Greater efficacy and increased range of action	Phage resistance, specificity and stability	Jia et al. (2023)
Combination therapies	Phage combinations with antibiotics and other therapies	Research on synergy and biofilm degradation	Improved treatment outcomes and reduced resistance	Resistance development, stability, safety, toxicity, regulatory approval, pharmacokinetics, cost, accessibility, and patient variability	Li et al. (2021)
Phage cocktails	Targeting various bacterial strains with combinations of phages	Spectrum of activity, formulation stability	Broad spectrum, enhanced efficacy, reduced resistance, improved treatment, complementary action	Phage compatibility, dosage complexity, resistance development, formulation stability, production costs, regulatory approval, identification and screening, interactions with host	Abedon et al. (2021)
Engineered phages	Designing genetically modified phages to enhance therapeutic properties	Genetic engineering, efficacy testing	More effective and targeted phage therapy	Safety concerns, off-target effects	Gibb et al. (2021)
CRISPR-Cas delivery	Using CRISPR-Cas systems to target and edit bacterial genomes	Target specificity, delivery methods, off-target effects, efficiency, safety, scalability, ethical considerations	Precision targeting of bacterial pathogens and reduced resistance	Delivery efficiency, immune response	Zhang et al. (2022)
Endolysins	Using endolysins to degrade the cell walls of bacteria	Enzyme activity, effectiveness against various bacteria	Enhanced bacterial cell lysis and treatment efficacy	Stability of enzymes, potential resistance	Rahman et al. (2021)
Phage behavior studies	Understanding phage dynamics in the human body	Pharmacokinetics, immune response studies	Safer and more predictable phage therapy outcomes	Complexity of human microbiome, individual variability	Navarro and Muniesa (2017)
Phage formulation development	Creating stable and effective phage formulations	Delivery methods, stability studies	Reliable and user-friendly phage products	Preservation of phage activity, cost	Rosner and Clark (2021)
Addressing phage resistance	Developing strategies to counteract phage resistance	Phage modification, resistance monitoring	Sustained efficacy of phage treatments	Evolution of bacterial defenses	Oromí-Bosch et al. (2023)

## Discussion and conclusion

Most of the findings support the use of phage therapy as an alternative treatment against multidrug-resistant bacteria such as *Enterococcus faecium, Staphylococcus aureus*, *Klebsiella pneumoniae*, *Acinetobacter baumannii*, *Pseudomonas aeruginosa*, and *Enterobacter spp*. These bacterial pathogens have narrow genetic diversity and are ideal targets for bacteriophages. Currently, phage therapy is not being used as a regular clinical practice due to the lack of sufficient clinical research required to understand its long-term effects and potential risks. Human trials have proven the safety of this therapy, but efficacy studies have shown mixed results. Some of the issues that must be addressed before this therapy can be widely used include overcoming the narrow host range of phages, systemic side effects, phage resistance, reduced immune system responses to phages, phage delivery issues, and challenges in phage manufacturing. To design optimal bacteriophage therapeutics, engineering phages is crucial. Engineered non lytic phages have the added advantage of reducing the levels of released endotoxin. Phage enzymes such as endolysin have effects on infectious Gram-positive bacteria. No resistance or development of neutralizing antibodies was observed. The combination of antibiotics and phages has resulted in low rates of resistance and increased bactericidal effects. To be able to use phage therapy as a regular clinical practice in the Western world, it is important to learn about the phage diversity that exists in nature and the interactions between phages and bacteria. Future studies and research should aim to test the efficacy of phages and their safety as well as the characterization and isolation of bacteriophages for use as tools against bacterial pathogens.

Phage therapy is an emerging and effective tool for resolving antibiotic resistance crises across the globe. It can be introduced in medicine and can be optimized for special treatments. It will help individual patients control and inhibit emerging bacterial epidemics such as chronic bacterial infections. It can be particularly useful in developing countries where bacterial diseases such as diarrhea are a major part of the infectious burden. Phage therapy can be used in combination with antibiotics or on its own depending on the patient’s situation. It is important to be more open to a sustainable ecological approach that allows the development, optimization, and implementation of phage therapy as a recognized scientifically meaningful approach for bacterial treatment. To make phage therapy, a commercially viable clinical treatment option, it is important to make approval standards more specific for phage therapy, which will help to accelerate the process of clinical approval while also maintaining safety regulations. Further research on safe administration and validation is required for the successful adoption of phage therapy in clinical settings.

## Conflict of interest

The authors declare that they have no conflicts of interest.

## Data Availability

Data will be available upon request to corresponding author.
